# Therapeutic efficacy of favipiravir against Bourbon virus in mice

**DOI:** 10.1371/journal.ppat.1007790

**Published:** 2019-06-13

**Authors:** Traci L. Bricker, Md. Shafiuddin, Anshu P. Gounder, Andrew B. Janowski, Guoyan Zhao, Graham D. Williams, Brett W. Jagger, Michael S. Diamond, Thomas Bailey, Jennie H. Kwon, David Wang, Adrianus C. M. Boon

**Affiliations:** 1 Division of Infectious Diseases, Department of Internal Medicine, Washington University School of Medicine, St Louis, Missouri, United States of America; 2 Department of Molecular Microbiology, Washington University School of Medicine, St Louis, Missouri, United States of America; 3 Department of Pathology and Immunology, Washington University School of Medicine, St. Louis, Missouri, United States of America; National Institute of Allergy and Infectious Diseases, UNITED STATES

## Abstract

Bourbon virus (BRBV) is an emerging tick-borne RNA virus in the *orthomyxoviridae* family that was discovered in 2014. Although fatal human cases of BRBV have been described, little is known about its pathogenesis, and no antiviral therapies or vaccines exist. We obtained serum from a fatal case in 2017 and successfully recovered the second human infectious isolate of BRBV. Next-generation sequencing of the St. Louis isolate of BRBV (BRBV-STL) showed >99% nucleotide identity to the original reference isolate. Using BRBV-STL, we developed a small animal model to study BRBV-STL tropism *in vivo* and evaluated the prophylactic and therapeutic efficacy of the experimental antiviral drug favipiravir against BRBV-induced disease. Infection of *Ifnar1*^-/-^ mice lacking the type I interferon receptor, but not congenic wild-type animals, resulted in uniformly fatal disease 6 to 10 days after infection. RNA *in situ* hybridization and viral yield assays demonstrated a broad tropism of BRBV-STL with highest levels detected in liver and spleen. *In vitro* replication and polymerase activity of BRBV-STL were inhibited by favipiravir. Moreover, administration of favipiravir as a prophylaxis or as post-exposure therapy three days after infection prevented BRBV-STL-induced mortality in immunocompromised *Ifnar1*^-/-^ mice. These results suggest that favipiravir may be a candidate treatment for humans who become infected with BRBV.

## Author summary

Bourbon virus (BRBV) is a novel tick-borne RNA virus that can cause fatal disease in humans. No approved antiviral treatment is available. We have cultured the second human isolate of BRBV and with it developed a small animal disease model. In this mouse model, BRBV causes severe disease as measured by weight loss after infection and uniform death 6 to 10 days after infection. Virus replication occurred predominantly in the spleen and the liver of the infected animals, with additional organs infected at later time points after infection. This disease model was used to test the efficacy of favipiravir, a viral RNA polymerase inhibitor that was developed for the related Influenza A virus. Prophylactic and therapeutic treatment with favipiravir resulted in complete protection from a lethal BRBV infection. These data suggest that favipiravir and perhaps other RNA polymerase inhibitors could be used to treat BRBV infections in humans.

## Introduction

Bourbon virus (BRBV) is an emerging tick-borne RNA virus and a member of the genus *Thogotovirus* in the family *orthomyxoviridae*. It was first discovered in 2014 in a clinical specimen obtained from a severely ill patient from Bourbon County, Kansas. This individual died two days later from complications of renal failure, acute respiratory distress syndrome, and ventricular tachycardia [1]. The genome of BRBV is composed of six gene-segments that are predicted to encode for PB2, PB1, and PA polymerase proteins, a nucleoprotein (NP), a surface glycoprotein (GP), and a matrix (M) protein. Following the initial discovery of this virus, one additional case of human BRBV infection was identified [2].

BRBV replicates in tick cell lines [3], and surveillance studies have identified BRBV-positive ticks near the border of Missouri and Kansas [2, 4]. All positive ticks reported to date belong to the species *Amblyomma americanum*, also known as the Lone-Star tick. *A*. *americanum* is an aggressive tick that feeds on many species including humans. The host range and natural reservoir of BRBV are not known, and antiviral therapies and vaccines against BRBV have not been developed. Here, we report on the isolation and characterization of the second human isolate of BRBV (BRBV-STL). BRBV-STL was cultured from a clinical specimen obtained from a fatal BRBV case. BRBV-STL replication was inhibited *in vitro* by the RNA polymerase inhibitor favipiravir. *In vivo* administration of favipiravir prior to or following BRBV infection protects against fatal disease in immunocompromised *Ifnar1*^-/-^ mice. These data support the evaluation of favipiravir as antiviral drug in future human cases of BRBV.

## Results

### Patient case

In June of 2017, a 58-year old Caucasian woman undergoing treatment for relapsed follicular lymphoma with rituximab and bendamustine presented with a two-week history of generalized weakness, myalgia, nausea, and rash, all of which occurred approximately one week after she noted exposure to ticks. She subsequently developed diarrhea and a fever of 39°C, prompting her admission to a hospital. Initial diagnostic laboratory workup was notable for marked thrombocytopenia (18,000 per mm^3^), leukopenia (2,400 per mm^3^), lymphopenia (500 per mm^3^), and mild elevation of aspartate transaminase (AST; 74 U/L); these abnormalities were not noted after previous cycles of her chemotherapy. Serological testing for Rocky Mountain spotted fever (RMSF) and HIV, as well as PCR testing for *Ehrlichia* and *Anaplasma* spp. from serum, were negative. The patient was started on empiric doxycycline and broad-spectrum antibiotics without clinical improvement. The patient continued to experience debilitating fatigue, progressive rash, and wheezing with mild hypoxia, although chest radiograph and chest computed tomography scan did not initially demonstrate pulmonary infiltrates. Her ferritin levels were high at 4,785 ng/mL, and systemic steroids were initiated due to concern for hemophagocytic lymphohistiocytosis (HLH). Bone marrow biopsy on hospital day 5 did not find evidence of lymphoma, but did show scattered hemophagocytic histiocytes. The patient’s rash progressed to include marked involvement of the palms and soles, with skin biopsy on hospital days 5 and 9 demonstrating interface dermatitis inconsistent with Rickettsial disease. Repeat RMSF serologies on hospital day 14 again were negative. In the setting of ongoing diarrhea, an ELISA for *Clostridium difficile* toxin was positive, and the patient was started on oral vancomycin. Notwithstanding these interventions, her diarrhea persisted, and she developed oral mucositis with ulcerations that were negative for herpes simplex virus by PCR. A serum sample sent to the Center for Disease Control, Division of Vector-Borne Diseases, on hospital day 3, was found to be positive for BRBV RNA on hospital day 10.

The patient’s wheezing and dyspnea worsened, and on hospital day 17, chest radiograph demonstrated new, diffuse, bilateral, upper lobe-predominant interstitial infiltrates with a small left-sided pleural effusion. She developed fluctuating cognitive impairment with disorientation and somnolence, and a brain MRI on hospital day 21 demonstrated mild white matter fluid-attenuated inversion recovery hyperintensity. Elevations in aspartate aminotransferase and alanine transaminase (482 and 110 U/L respectively on hospital day 21) and ferritin (11348 ng/mL) worsened, prompting the initiation of etoposide to treat HLH. The patient’s mental status and hypoxic respiratory failure continued to worsen, and she was transferred to the intensive care unit. A transthoracic echocardiogram showed left ventricular systolic dysfunction and a hemorrhagic pericardial effusion. She died on hospital day 23.

### Isolation and characterization of BRBV-STL

**Fig 1:**
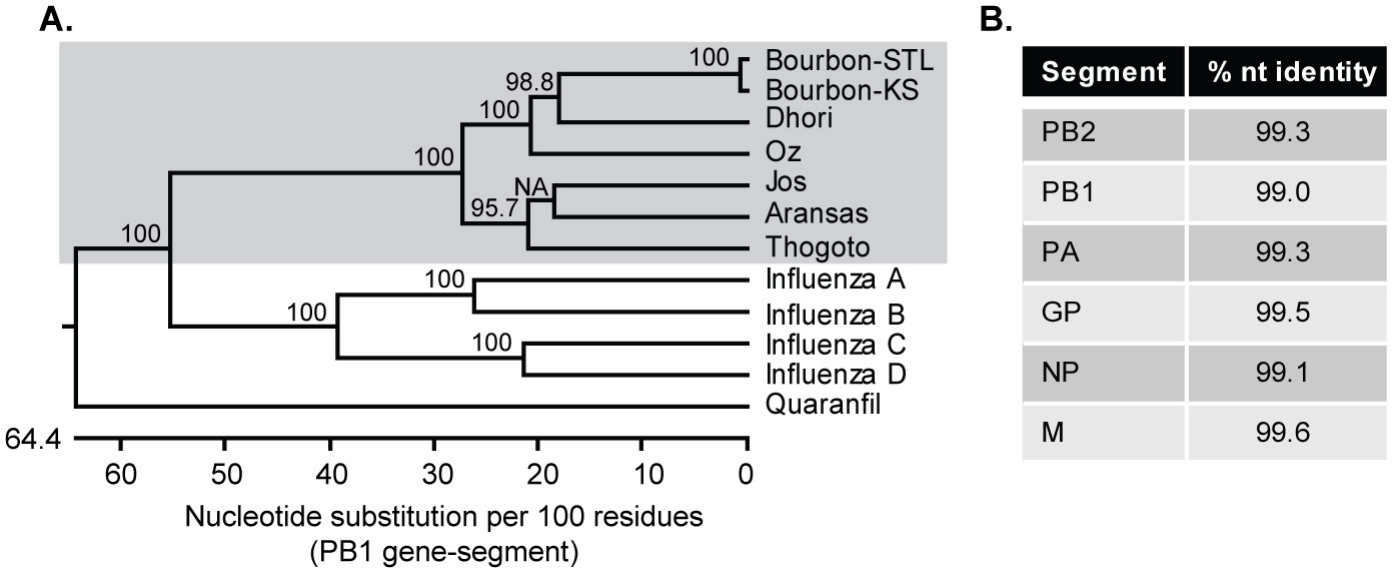
Molecular characterization of BRBV-STL. BRBV-STL was identified in a blood sample from a hospitalized patient. The genome sequence of the virus was determined by next-generation sequencing on RNA extracted from culture supernatant of Vero cells inoculated with the patient serum. (**A**) Phylogenetic tree of segment 2 (PB1 gene) of different orthomyxoviruses. The tree was constructed using ClustalW in the DNASTAR Lasergene 15 software package. Bootstrap values were calculated on a 1000 trails and the values are included in the figure. NA, no value available. Bourbon virus strain St. Louis (BRBV-STL, MK453528); Bourbon virus strain original (BRBV-KS, KU708254.1); Dhori virus (NC_034263.1); Oz virus (LC320124.1); Jos virus (HM627170.1); Aransas virus (KC506163.1); Thogoto virus (AF004985.1); Influenza A virus (CY009450); Influenza B virus (AY582058.1); Influenza C virus (NC_006308.2); Influenza D virus (LN559121.1); Quaranfil virus (FJ861695.1). BRBV-STL belongs to the *Thogotovirus* genus (grey background) and is very similar to the original strain of BRBV. (**B**) Percent identity at the nucleotide (nt) level between our BRBV isolate (BRBV-STL) and the original BRBV isolate (BRBV-KS).

Serum collected from the patient was used to inoculate Vero cells. Three days later, cytopathic effect (CPE) was observed in the cells inoculated with a 1:5 dilution of the serum. CPE was observed 4 and 5 days post infection (dpi) in the cells inoculated with 1:50 and 1:500 dilution of serum. RNA was extracted from the supernatant of these infected cells and used for next generation sequencing; the resulting sequence identified BRBV. The average sequence coverage was 10,000x with lower coverage near the 3’ and 5’ untranslated regions of each gene-segment. Compared to the original isolate of BRBV from Kansas (BRBV-KS) [1, 3], BRBV-STL is 99.3% identical at the nucleotide level (Fig 1). A total of 21 amino acid differences were identified in five of the six predicted viral proteins (Table 1). Of note, one region in the PB1 protein (573–577) and another in the NP (67–71) protein were variable between the two BRBV isolates.

**Table 1:** Amino-acid differences between the St. Louis (BRBV-STL) and original isolate of BRBV (BRBV-KS).

Gene-segment	Amino acid variation
PB2	N22S, K289R, I376V, K763R
PB1	R81K, S558G, C573S, S574I, G575S, I577L
PA	I34V, K207R, T263N, R555K
GP	N35D, I63V
NP	T67S, L68I, C69V, L70M, S71A
M	No amino-acid changes

### Favipiravir has antiviral activity in vitro

**Fig 2:**
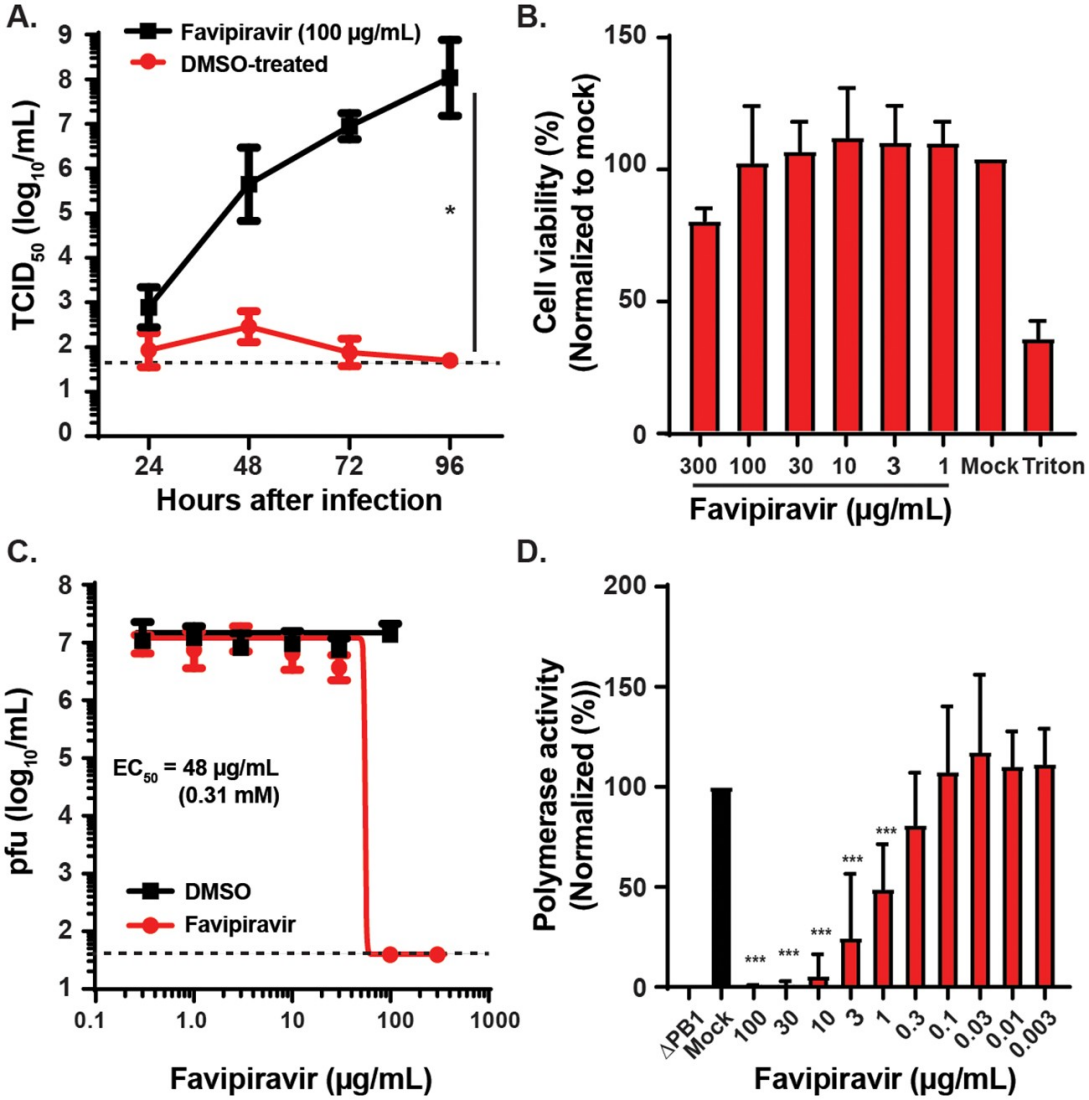
Favipiravir inhibits BRBV-STL replication and polymerase activity. (**A**) Vero cells were inoculated with 20 pfu of BRBV-STL in the presence or absence of 100 μg/mL (0.64 mM) favipiravir. The virus titer in the culture supernatant was quantified every 24 h for 4 days. Values are means (± standard deviation) of the virus titer from two experiments performed in duplicate. *, *P <* 0.05 by Mann-Whitney U-test on the slope of the curves fit by linear regression of ln-transformed virus titer over time. The dotted line represents the limit of detection at 50 TCID_50_/mL. (**B**) XTT assay to measure Vero cell viability at different doses of favipiravir or solvent (DMSO). The results are normalized to untreated (Mock) control cells. Triton X-100 (1%, Triton) was used as a positive control. Values are the means (+ standard deviation) of the normalized cell viability data. Each condition was tested in triplicate, and each experiment was repeated three independent times. (**C**) Vero cells were inoculated with 20 pfu of BRBV-STL in the presence of different concentrations of favipiravir or DMSO (solvent), starting at 300 μg/mL (1.9 mM). The virus titer in the culture supernatant was quantified by plaque assay at 3 dpi. Values are geometric means (± standard deviation) of the virus titer from three experiments performed in duplicate. A curve was fitted through the data using the log(inhibitor) vs. response—variable slope equation (GraphPad Prism 8.0). The dotted line represents the limit of detection at 40 pfu/mL. (**D**) The effect of different concentrations of favipiravir on the polymerase activity was quantified in a mini-genome reporter assay. Expression plasmids encoding for the PB2, PB1, PA and NP of BRBV-STL and Renilla luciferase, plus the reporter construct (Firefly luciferase flanked by the 3' and 5' UTR of segment 5 of BRBV-STL) were transfected into 293T cells in the presence or absence of different concentrations of favipiravir. Three days post transfection the amount of firefly luciferase was quantified and normalized across wells and conditions using the Renilla luciferase data. Values are the means (+ standard deviation) of the normalized polymerase activity from seven experiments performed in duplicate. ***, *P <* 0.001 by one-way ANOVA correcting for multiple comparisons.

Favipiravir is a drug with efficacy against influenza A virus (a distantly related *orthomyxoviridae* family member) that acts by inhibiting its RNA dependent RNA polymerase [5–7]. Since BRBV is a member of the *orthomyxoviridae*, we hypothesized that favipiravir might have antiviral activity against BRBV. To test this, we performed a virus growth assay in the presence or absence of 100 μg/mL of favipiravir and measured viral titers in the culture supernatant every 24 hours (h) for 4 days by a virus titration assay (TCID_50_/mL). Without favipiravir, BRBV-STL grew to ~10^8^ TCID_50_/mL in 4 days. In contrast, addition of the drug immediately after infection inhibited virus production in the culture supernatant nearly a million-fold (Fig 2a). At this dose of favipiravir, the viability of the Vero cells was minimally affected (Fig 2b).

Next, we determined the minimal effective concentration (EC_50_) of favipiravir against BRBV-STL. Vero cells were infected with BRBV-STL in the presence of different concentrations of favipiravir. Culture supernatant was collected at 72 hours post infection (hpi) and the virus titer was quantified by plaque assay (pfu/mL). No replicating virus was detected by plaque assay at 100 and 300 μg/mL (0.64 and 1.9 mM) concentrations of favipiravir (Fig 2c); concentrations that did not affect the viability of Vero cells (Fig 2b). No inhibitory effect of favipiravir on BRBV-STL virus titers was observed at 30 μg/mL or lower concentrations (Fig 2c). Based on this data, the EC_50_ of favipiravir against BRBV was calculated as ~48 μg/mL (0.31 mM).

To confirm that favipiravir targets the polymerase complex of BRBV-STL, we developed a mini-genome reporter assay for BRBV-STL. Transfection of 293T cells with expression plasmids encoding for the PB2, PB1, PA and NP proteins, plus the firefly-luciferase reporter gene, resulted in robust firefly-luciferase activity (Fig 2d). This activity was specific for the polymerase complex of BRBV, as removal of the essential polymerase gene PB1 resulted in no reporter activity. This assay was used to quantify the inhibitory effects of favipiravir on BRBV replication. The polymerase activity of BRBV-STL was significantly reduced at 100, 30, 10, 3 and 1 μg/mL (640–6.4 μM) concentrations of favipiravir compared to the mock-treated control (*P <* 0.001, Fig 2d); the EC_50_ value was calculated as 1 μg/mL (6.4 μM).

### Development of a BRBV mouse model

**Fig 3:**
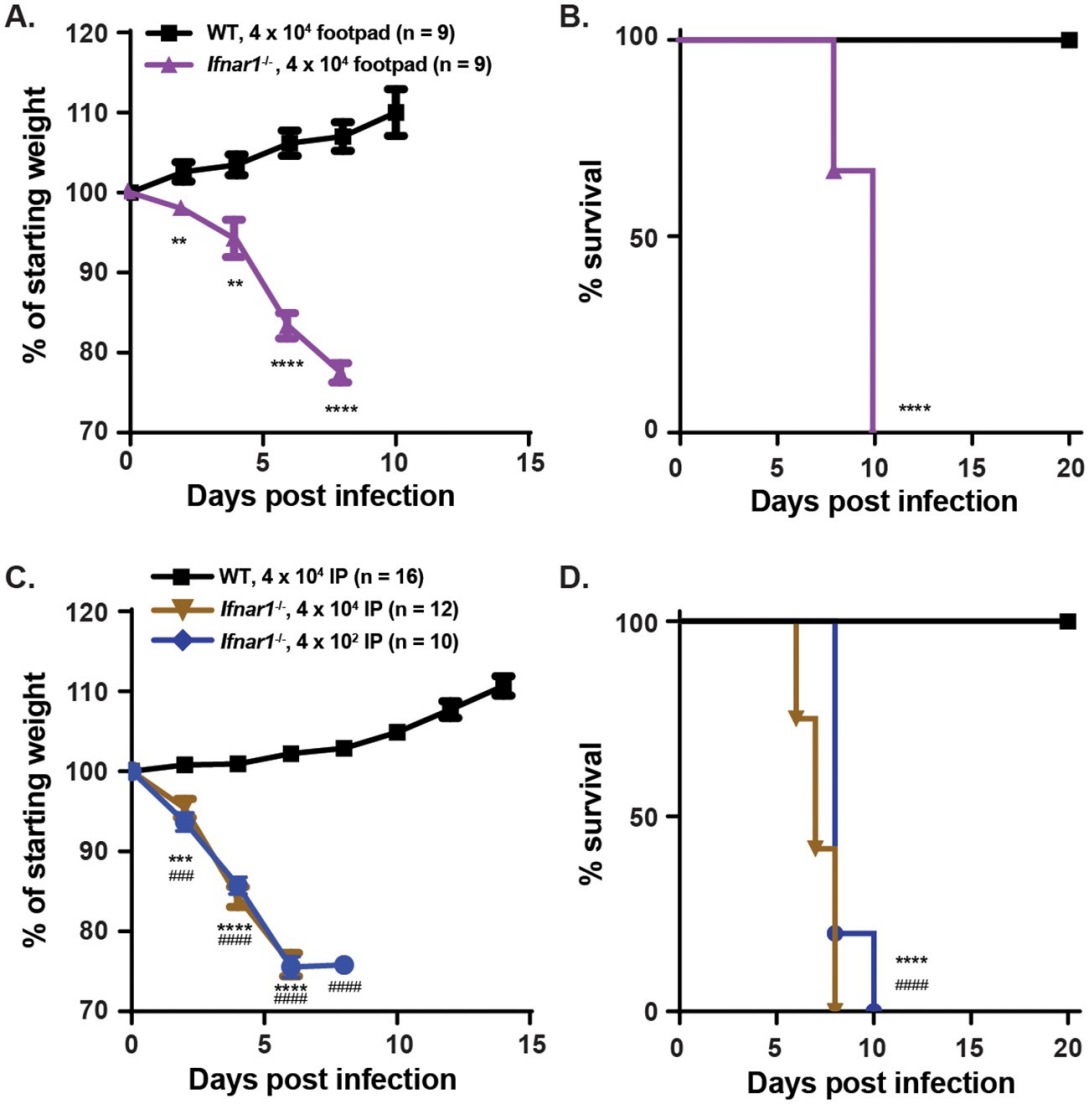
Mouse model of BRBV disease. (**A and B**) WT (n = 9), and *Ifnar1*^-/-^ mice (n = 9), were inoculated with 4 x 10^4^ pfu of BRBV-STL in the footpad. Weight change (**A**) and survival (**B**) were monitored for 10 and 20 days respectively. Values are the means (± standard error of the mean) from two experiments. Weight change is analyzed by an independent t-test and survival by the log-rank test. **, *P <* 0.01; ****, *P <* 0.0001. (**C and D**) WT and *Ifnar1*^-/-^ mice were inoculated via intraperitoneal route with 4 x 10^4^ (n = 16 for WT and n = 12 for *Ifnar1*^-/-^) or 4 x 10^2^ (n = 10 for *Ifnar1*^-/-^) pfu of BRBV-STL and weight change (**C**) and survival (**D**) were monitored for 14 and 20 days respectively. Values are the means (± standard error of the mean) from two or more experiments. Weight change is analyzed by an independent t-test and survival by the log-rank test. ***, *P <* 0.001, ****, *P <* 0.0001 between WT and *Ifnar1*^-/-^ mice at 4 x 10^4^ pfu. ^###^, *P <* 0.001; ^####^, *P <* 0.0001 between WT at 4 x 10^4^ pfu and *Ifnar1*^-/-^ mice at 4 x 10^2^ pfu.

Subcutaneous inoculation of wild-type WT mice with 4 x 10^4^ plaque forming units (pfu) of BRBV-STL in the footpad resulted in no weight loss or mortality after infection (Fig 3a and 3b). As replication of human viruses in mice often is inhibited by type I interferon (IFN) signaling and induction of antiviral gene expression due to a species-specific failure to antagonize this key host antiviral pathway [8], we repeated experiments in *Ifnar1*^-/-^ mice lacking the type I IFN receptor. Footpad inoculation of *Ifnar1*^-/-^ mice with 4 x 10^4^ pfu of BRBV-STL resulted in significant weight loss (*P <* 0.0001), starting at 4 dpi and 100% mortality by 10 dpi (*P <* 0.0001, Fig 3a and 3b). A similar result was observed after inoculation of 4 x 10^4^ pfu of BRBV-STL via the intraperitoneal route (Fig 3c and 3d). All *Ifnar1*^-/-^ mice started losing weight at 2 dpi and succumbed to infection at 8 dpi. Intraperitoneal (IP) inoculation with 4 x 10^2^ pfu of BRBV-STL also resulted in substantial weight loss (*P <* 0.0001) and 100% mortality after infection (*P <* 0.0001, Fig 3c and 3d).

### Tissue tropism of BRBV-STL in Ifnar1^-/-^ mice

**Fig 4:**
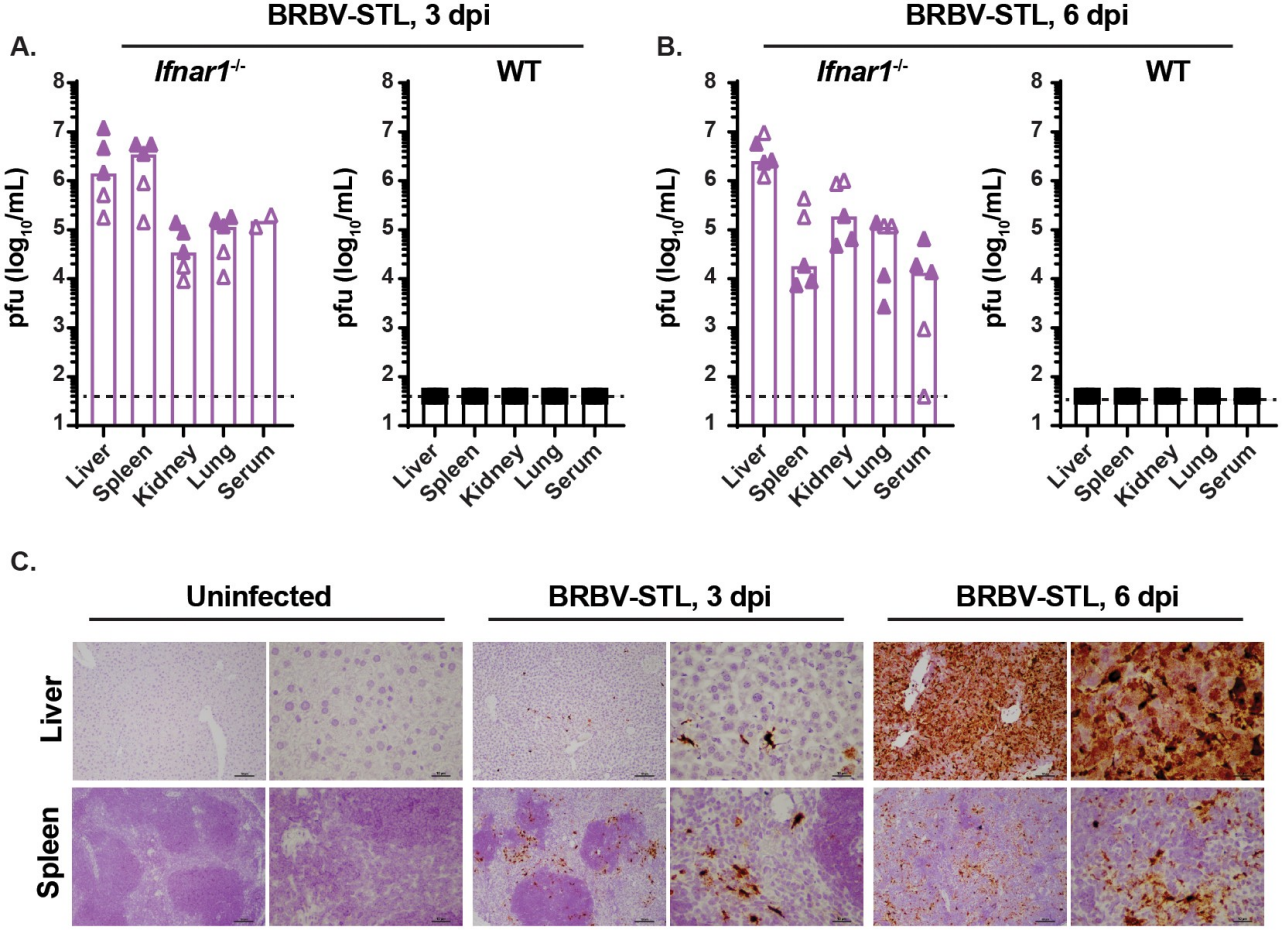
Liver and spleen tropism of BRBV-STL in *Ifnar1*^-/-^ mice. (**A and B**) Virus titers (pfu/mL) were quantified in the liver, spleen, kidney, lung and serum of WT and *Ifnar1*^-/-^ mice 3 and 6 dpi after IP inoculation with 4 x 10^4^ pfu of BRBV-STL. Tissues were collected, homogenized, and the virus titer was determined by plaque assay. Each data point is a single mouse obtained from two different experiments (indicated by the open and closed symbols). The bar represents the median virus titer observed in each of the tissues. Viremia in serum at 3 dpi was obtained from two mice in one experiment. The dotted line represents the limit of detection of the assay at 40 pfu/mL. (**C**) RNA *in situ* hybridization on sections from liver and spleen from uninfected of BRBV-STL infected (4 x 10^4^ pfu of BRBV-STL via IP) *Ifnar1*^-/-^ mice. Probes targeting segment 5 of BRBV were used to visualize BRBV-infected cells, indicated by the dark brown staining. Sections were counterstained with hematoxylin prior to mounting and analysis. From left to right are uninfected, 3 dpi and 6 dpi at 10x and 40x magnification. Top panel are liver sections and the bottom panel are spleen sections. The images are representative of sections obtained from three mice in one experiment.

To characterize the tissue tropism of BRBV-STL, we collected serum, liver, spleen, kidney, and lung tissues at 3 and 6 dpi from WT and *Ifnar1*^-/-^ mice inoculated with 4 x 10^4^ pfu of BRBV-STL via intraperitoneal route. The tissues were homogenized and the viral load was quantified by plaque assay on Vero cells. At 3 dpi, we observed high virus titers (10^5^–10^7^ pfu/mL) in the liver and spleen of *Ifnar1*^-/-^ mice, whereas the serum, kidneys, and lungs of *Ifnar1*^-/-^ mice had lower virus titers (10^3^–10^5^ pfu/mL) (Fig 4a). In contrast, WT mice had no detectable virus in all of the organs tested. At 6 dpi, the liver and spleen of BRBV-STL inoculated *Ifnar1*^-/-^ mice still contained high levels of virus, although the titer in the spleen had dropped by ~100-fold (Fig 4b). No replicating virus was detected in WT mice at this time point.

To further define the tropism of BRBV-STL in *Ifnar1*^-/-^ mice, we performed RNA *in situ* hybridization (RNA-ISH) and histological analysis on tissue sections of BRBV-STL infected wild type and *Ifnar1*^-/-^ mice (Fig 4c, S1 and S2 Figs). At 3 dpi, viral RNA was detected in the liver and spleen of all three *Ifnar1*^-/-^ mice. In the liver, the staining corresponded to sinusoidal cells, whereas in the spleen viral RNA was primarily detected in macrophages of the white pulp with fewer positive cells in the red pulp. One animal showed minimal staining in the lungs that corresponded to alveolar macrophages. No viral RNA was detected in the brain, heart, and kidney at 3 dpi. At 6 dpi, viral RNA was detected in liver, spleen, lung, heart and kidney of all three *Ifnar1*^*-/-*^ mice (Fig 4c, and S2 Fig). Only one of the mice was positive for viral RNA in the brain. Staining of these same tissues with hematoxylin and eosin (H & E) revealed minimal multifocal hepatitis with signs of inflammation at 3 dpi, and moderate diffuse hepatitis with coagulative necrosis and extra medullary granulopoiesis in the liver of *Ifnar1*^-/-^ mice (S1 Fig). In the white pulp of the spleen there was mild to moderate lymphoid necrosis and inflammation at 3 dpi. At 6 dpi, there was marked diffuse lymphocyte necrosis and inflammation in the white pulp and an increase in extramedullary granulopoiesis in the red pulp. Finally, in the heart and lungs there was evidence of neutrophil and mononuclear cell infiltration at 6 dpi. No significant lesions were found in the kidneys and brains of *Ifnar1*^-/-^ mice at 6 dpi. In contrast, no BRBV RNA positive cells were detected in any of the tissue sections from WT mice (S2 Fig).

### Favipiravir protects mice from lethal BRBV-STL infection

**Fig 5:**
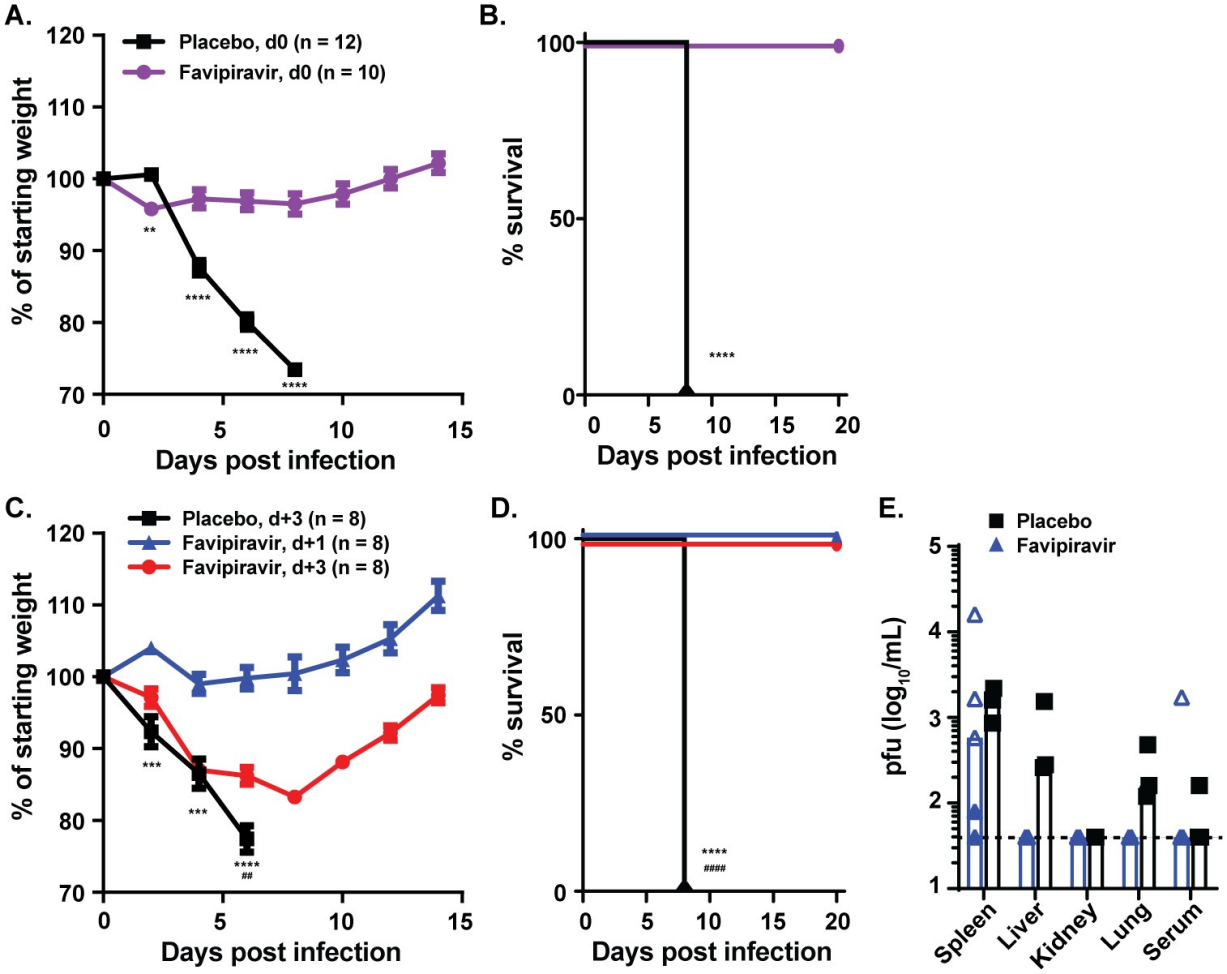
Favipiravir protects mice from fatal BRBV infection. (**A and B**) *Ifnar1*^-/-^ mice were treated with 150 mg/kg of favipiravir in 0.5% methylcellulose twice daily per oral gavage (n = 10) or 0.5% methylcellulose alone (n = 12) for eight days starting 2 hours prior to infection with 4 x 10^2^ pfu of BRBV-STL via IP. Weight change (**A**) and survival (**B**) were monitored for 14 and 20 days respectively. Values are the means (± standard error of the mean) from three experiments. Weight change is analyzed by an independent t-test and survival by the log-rank test. **, *P <* 0.01; ****, *P <* 0.0001. (**C and D**) *Ifnar1*^-/-^ mice were treated eight days with 150 mg/kg of favipiravir in 0.5% methylcellulose twice daily starting one day (d+1, blue symbols, n = 8) or three days (d+3, red symbols, n = 8) after IP inoculation with 4 x 10^2^ pfu of BRBV-STL. Mice receiving 0.5% methylcellulose alone for eight days starting three days after inoculation (placebo d+3, n = 8) were used as controls. Weight change (**C**) and survival (**D**) were monitored for 14 and 20 days respectively. Values are the means (± standard error of the mean) from two experiments. Weight change is analyzed by an independent T-test and survival by the log-rank test. ***, *P <* 0.001; ****, *P <* 0.0001 between placebo and d+1 treated animals; ^##^, *P <* 0.01, ^####^, *P <* 0.0001 between placebo and d+3 treated animals. (**E**) Virus titers (pfu/mL) were quantified in various organs of mice infected with 4 x 10^2^ pfu of BRBV-STL and treated one day after infection with 150 mg/kg of favipiravir twice daily for three days (n = 5) or 0.5% methylcellulose alone (n = 3). Each data point is a single mouse obtained from two different experiments (indicated by the open and closed symbols). The bar represents the median virus titer observed in each of the tissues. The dotted line represents the limit of detection of the assay at 40 pfu/mL.

To test whether favipiravir had efficacy against BRBV-STL *in vivo*, we inoculated *Ifnar1*^-/-^ mice via IP route with 4 x 10^2^ pfu of BRBV-STL and treated the mice twice daily with 150 mg/kg of favipiravir in 0.5% methylcellulose via oral gavage for 8 days beginning immediately after infection. Compared to mock-treated animals, favipiravir treated animals did not show evidence of weight loss (*P <* 0.0001) after infection, and none of the treated animals succumbed to infection (*P <* 0.0001, Fig 5a and 5b). We next evaluated the therapeutic effect of favipiravir. Animals treated twice daily with 150 mg/kg of favipiravir for 8 days starting 1 dpi did not lose weight, and all animals survived infection (Fig 5c and 5d). Analysis of the virus burden in different organs revealed low to undetectable BRBV replication 3 days after initiation of treatment (Fig 5e) in liver and lungs of the treated mice. In comparison, mock-treated mice had demonstrable virus titers in these organs (Fig 5e). No effect of favipiravir on spleen virus load was detected. To determine if favipiravir was effective after the onset of symptoms, we initiated treatment at 3 dpi with BRBV-STL. At this time point, the animals were starting to lose weight and replicating BRBV-STL was detected throughout the body (Fig 4a). One-day after favipiravir treatment initiation (4 dpi), the mice stopped losing weight and gradually recovered from the infection (Fig 5c). Remarkably, all of the animals survived the infection, whereas the mock-treated animals all succumbed (*P <* 0.001, Fig 5d). Collectively, these data show that favipiravir treatment has therapeutic efficacy against BRBV-STL in this pre-clinical mouse model.

## Discussion

BRBV is a recently discovered tick-borne orthomyxovirus that was first identified in 2014 in a human patient who died due to complications from the disease. Currently, there is no approved therapy or vaccine against BRBV. Here we report the isolation and characterization of the second human isolate of BRBV (BRBV-STL) from a fatal case and demonstrate that BRBV replication is inhibited by the broad-spectrum antiviral drug favipiravir. Using a novel mouse model of BRBV pathogenesis, we also show that favipiravir has prophylactic and therapeutic activity against fatal BRBV disease. These findings support the possible evaluation of favipiravir as a therapeutic for future human BRBV cases.

This study describes a lethal mouse model for BRBV. A previous study showed that immunocompetent mice inoculated via IP or intracranial routes with BRBV sustained no signs of morbidity or mortality [3]. We confirmed this finding, as we observed no signs of weight loss or death after inoculation with high doses of BRBV-STL (4 x 10^4^ pfu). Partial neutralization of the type I IFN response, using a blocking monoclonal antibody against the type I IFN receptor (MAR1-5A3) did not result in fatal BRBV disease (S3 Fig). However, some replicating virus was detected 3 dpi in the spleen of mice suggesting that inhibition of the innate antiviral immune response increased the susceptibility to BRBV infection. Indeed, inoculation of *Ifnar1*^-/-^ mice with low doses of BRBV-STL resulted in high viral titers in liver and spleen, weight loss, and uniform death 7–10 days after infection. *Ifnar1*^-/-^ mice have been used previously for other viral animal models, including Ebola, Zika, West Nile (WNV) and severe fever with thrombocytopenia syndrome (SFTSV) viruses [9, 10]. Viral pathogenesis in *Ifnar1*^-/-^ mice has been associated with higher viral burden and increased inflammation caused by the dysregulation of type I IFN inducible negative regulation of inflammation [11, 12]. In the WNV and SFTSV models in *Ifnar1*^-/-^ mice, the inflammatory dysregulation results in uniform death of the mice at 3 and 4–5 dpi, respectively. It is not known if BRBV-STL induces a similar dysfunctional inflammatory response, and if this results in demise of the mice. A comparison between the pathophysiology of BRBV disease in mice and humans is difficult to make given the small number of human cases reported to date. BRBV-STL demonstrated broad tissue tropism in immunocompromised *Ifnar1*^-/-^ mice; particularly high levels of viral replication were noted in the liver and spleen, but substantial quantities of BRBV RNA were also detected in lung, serum and kidney. In both cases of human BRBV disease reported to date, a systemic illness progressing to multiple organ dysfunction was noted, and viremia was present. Which of these multisystem manifestations of BRBV disease is directly attributable to viral replication in the relevant end organs is unclear, as systemic inflammation could accompany BRBV infection and also cause disease. Indeed, the presence of hemophagocytic histiocytes in the bone marrow biopsy is suggestive of a systemic inflammatory response to viral infection. However, the elevations in serum ALT, AST, and ferritin levels in both human cases reported to date are also consistent with cytopathic BRBV replication in the liver, as was seen in *Ifnar1*^-/-^ mice.

Favipiravir is broad-spectrum antiviral compound that inhibits the RNA dependent RNA polymerase of RNA viruses [13, 14]. It was originally discovered as an antiviral compound against influenza A viruses (IAV), but subsequent studies showed a broad-spectrum activity against other RNA viruses. Since BRBV is a member of the family of *orthomyxoviridae*, like IAV, we hypothesized that favipiravir might have antiviral activity against BRBV. Our *in vitro* studies show that favipiravir inhibits BRBV replication; however, the EC_50_ is significantly higher compared to IAV (0.03–1.6 μg/mL) [15, 16]. The basis for this difference is not known, but it is possible that the amino acid differences between the viruses affect the shape or size of the binding pocket in the PB1 protein and therefore reduces the EC_50_. Structural studies and comparisons between various PB1 proteins may identify the reason for this reduced sensitivity. Multiple antiviral compounds against influenza virus are in clinical trials, and many of these compounds target components of the polymerase complex (PB2, PB1 and PA proteins) of Influenza A virus. It will be important to test these anti-influenza virus drugs against BRBV to identify additional inhibitors of virus replication and perhaps develop a combinatorial therapy to increase effectiveness and avoid resistance [17].

The EC_50_ value of favipiravir against BRBV-STL in Vero cells was relatively high and comparable to that of Ebola and yellow fever viruses [14]. Despite this, administration of 150 mg/kg favipiravir twice daily three days after BRBV-STL infection, when virus infection was established in many different organs, was 100% effective, and all of the animals survived infection. The serum concentration of favipiravir following 150 mg/kg twice daily administration reaches 200 μg/mL concentration [16], which is well above the EC_50_ value measured in Vero cells, and 100-fold higher than the EC_50_ value in the mini-genome reporter assay. Future titration studies will identify the minimal effective dose of favipiravir in this model and evaluate if combinations of antiviral drugs, such as a polymerase inhibitor and an endonuclease inhibitor can increase the efficacy of either compound alone.

The effective concentration (EC_50_) of favipiravir in the mini-genome assay is nearly 100-fold lower than the virus replication assay. This difference is not caused by a change in cell type, since the EC_50_ for virus replication on 293T cells was similar than that of Vero cells (S4 Fig). It is possible that the mini-genome assay is more sensitive to perturbation of PB1 activity, the target of favipiravir, or the assay conditions increase intracellular drug concentrations. Alternatively, the expression of the other BRBV proteins inhibit the activity, transport, or activation of the drug.

The genome of BRBV-STL is highly similar to that of the original isolate, BRBV-KS, with the exception of two regions that distinguish the viruses. These two regions are in the PB1 and NP protein, and the significance of these changes is not known. The amino acid differences in the PB1 and NP protein between BRBV-KS and BRBV-STL also were found in RNA directly obtained from the clinical specimen excluding the possibility of tissue culture adaptations. Since BRBV-STL and BRBV-KS were isolated from geographically distinct areas, it is possible that the differences in genome sequence are caused by the differences in host or tick species between these two geographic areas.

In summary, our study has shown that the pan antiviral compound favipiravir can treat an ongoing BRBV infection in a pre-clinical animal model, suggesting that favipiravir may be a candidate drug for the treatment of BRBV in humans.

## Material and methods

### Human studies

This study was approved by the Human Research Protection Office of Washington University School of Medicine in St. Louis. Informed consent was obtained.

### Animals and cells

Four to eight-week-old male and female C57BL/6J (WT) and *Ifnar1*^-/-^ mice were bred in a barrier facility at Washington University School of Medicine. All animal studies were approved and performed in accordance with the Washington University School of Medicine Institutional Animal Care and Use Committees. Vero cells (ATCC) were grown in Dulbecco’s Modified Eagle Medium (DMEM) media (Corning Cellgro) supplemented with 10% Fetal Bovine Serum (FBS, Biowest), 100 U/mL Penicillin (Life Technologies), 100 μg/mL streptomycin (Life Technologies), and 2 mM L-Glutamine (Corning). 293T cells (gift from Dr. Webby at St. Jude Children’s Research Hospital) were maintained in Opti-MEM (Life Technologies) with 10% Hyclone FBS (Thermo Fisher Scientific), 2 mM L-glutamine, 100 U/ml of Penicillin and 10 μg/mL of Streptomycin.

### Virus

Bourbon virus (BRBV) was isolated from a serum sample obtained from a 58-year old female who was admitted to Barnes Jewish Hospital in St. Louis in June of 2017. This isolate, BRBV-STL, grew to high titers in Vero cells, similar to that reported for the reference isolate [3]. A passage 2 (P2) stock of BRBV-STL was aliquoted, stored at -80°C, and used for all subsequent studies. The virus titer of the P2 stock was 2 x 10^8^ TCID_50_/mL or 4 x 10^7^ pfu/mL.

### Virus genome sequencing

RNA was isolated from the supernatant of Vero cells inoculated with the serum of the patient using the E.Z.N.A. Total RNA kit (Omega Bio-tek). The RNA was used to identify the genome sequence and clone segments 3–6 into expression vectors. For next-generation sequencing (NGS), the sample was amplified using a random RT-PCR protocol, and the resulting amplicons were used to generate a library using the NEBNext (NEB) kit as described [18]. The library was sequenced on the 2x250 base pair Illumina MiSeq platform. We used BWA to align the reads and LoFreq version 2 to identify SNPs relative to the published sequence of the original isolate of BRBV (BRBV-KS) [19, 20].

### Phylogenetic analysis

A phylogenetic tree of segment 2 (PB1 gene) of different orthomyxoviruses was constructed using ClustalW in the DNASTAR Lasergene 15 software package. Bourbon virus strain St. Louis (BRBV-STL, MK453528); Bourbon virus strain original (BRBV-KS, KU708254.1); Dhori virus (NC_034263.1); Oz virus (LC320124.1); Jos virus (HM627170.1); Aransas virus (KC506163.1); Thogoto virus (AF004985.1); Influenza A virus (CY009450); Influenza B virus (AY582058.1); Influenza C virus (NC_006308.2); Influenza D virus (LN559121.1); Quaranfil virus (FJ861695.1).

### Virus titration assay

The virus titer in stocks, supernatants and organs was quantified by plaque assay (pfu/mL) or virus titration assay (TCID_50_/mL). For the plaque assay, confluent monolayers of Vero cells were grown in 24-well plates overnight. The next day, the cells were washed with serum free DMEM medium and inoculated with 250 μL of medium containing 10-fold serially diluted virus, culture supernatant, or organ homogenate, starting at a 1:10 dilution. After 1 h at 37°C, the inoculum was aspirated and 1 mL of 2% methylcellulose in DMEM supplemented with 2% FBS, 100 U/mL Penicillin, 100 μg/mL streptomycin, and 2 mM L-Glutamine was added to each well. The plate was incubated for 6 days at 37°C/5% CO_2_ before the monolayer was fixed with 250 μL of 4% paraformaldehyde (PFA, in PBS) for 1 h and stained with 0.5% crystal violet for 1 h at 20°C. The number of plaques per dilution were enumerated and used to calculate the pfu/mL. For the TCID_50_ virus titration assays, confluent monolayers of Vero cells were grown in 96-well plates overnight. The next day, the cells were washed with serum-free DMEM medium and inoculated with 100 μL of DMEM medium containing 10-fold serially diluted virus, culture supernatant, or organ homogenate, starting at a 1:10 dilution. After 1 h at 37°C/5% CO_2_, the inoculum was aspirated and 200 μL of fresh DMEM supplemented with 2% FBS, 100 U/mL Penicillin, 100 μg/mL streptomycin, and 2 mM L-Glutamine (D2F media) was added to each well, and the plate was incubated for 6 days at 37°C. Next, the cells were fixed with 4% PFA for 1 h and stained with 0.5% crystal violet at 20°C. The cytopathic effect from BRBV destroys the monolayer and this can be used to quantify virus load according to the Reed & Munch method.

### Favipiravir inhibition assay

Confluent monolayers of Vero cells in 24-well plates were inoculated with 20 pfu (Multiplicity of infection (MOI) = 0.001) for 1 h at 37°C/5% CO_2_. Next, the inoculum was aspirated and the cells were washed with medium before 1.0 mL of fresh D2F was added to each well. To test the effects of favipiravir, different concentrations (300 μg/mL to 1 μg/mL) of the compound, diluted in DMSO, were added to the wells. Each concentration of favipiravir was tested in duplicate per experiment and the experiment was repeated three times. Control wells were treated with the same concentration of DMSO. The 300 μg/mL and 100 μg/mL concentration of favipiravir have the same DMSO control (1% final concentration). Culture supernatant was collected at different time points and used to quantify the amount of infectious virus produced by virus titration assay.

### Cytotoxicity assay

The cytotoxicity of favipiravir on 293T and Vero cells was evaluated by 2,3-Bis(2-methoxy-4-nitro-5-sulfophenyl)-2H-tetrazolium-5-carboxanilide (XTT) assay. Cells were seeded at 4 x 10^4^ cells per well in a 96-well plate. After overnight incubation, different concentrations of favipiravir diluted in RPMI 1640 media (Gibco) were added to each well. Cells treated with 1% Triton X-100 were used as the positive control for cell death. After 3 days, cells were treated with 0.2 mg/mL XTT and 0.1 mM phenazine methosulfate for 2 h at 37°C. XTT reduction to formazan was measured at 450 nm wavelength and background (630 nm) corrected absorbance values were used to calculate cell viability.

### BRBV reporter assay

The PB2, PB1, PA, and NP genes of BRBV-STL were cloned into pcDNA3.1+ expression vectors. The PA and NP genes were derived from cDNA generated from BRBV-STL RNA extracted from Vero cell culture supernatant inoculated with a 1:5 dilution of the patient serum. The PB2 and PB1 genes of BRBV-STL were synthesized by GENEWIZ. The firefly luciferase gene was cloned into the pLuci vector flanked by the 3' and 5' untranslated region of segment 6 of the original BRBV isolate (BRBV-KS). A total of 500 ng of DNA split evenly between the 4 BRBV genes, the firefly reporter construct and the Renilla luciferase internal control was transfected into 293T cells in 24-well plates using TransIT LT1 (Mirus Bio). Two days later, the amount of firefly and Renilla luciferase activity were quantified with the Dual luciferase assay reporter system (Promega). To test the effects of favipiravir, different concentrations of the compound dissolved in DMSO were added to the well along with the transfection mixture. The polymerase activity for each condition was normalized to that of the mock-treated (DMSO only) control cells.

### Mouse experiments

WT and *Ifnar1*^-/-^ mice were inoculated subcutaneously with 4 x 10^4^ pfu of BRBV-STL via footpad injection, or with 4 x 10^2^ or 4 x 10^4^ pfu via intraperitoneal injection. Weight change and survival were monitored for 14 days. To identify tissue tropism, organs from BRBV-STL infected animals were harvested 3 and 6 dpi, and viral load was determined by plaque assay on Vero cells after homogenization in 1.0 mL of DMEM media. For RNA *in situ* hybridization, organs from BRBV-STL infected or mock-infected animals were harvested 3 and 6 dpi and fixed in 10% formalin for 7 days prior to paraffin embedding and sectioning. To evaluate the prophylactic and therapeutic activity of favipiravir (BOC Sciences, 259793-96-9) against BRBV-STL, mice were inoculated via the intraperitoneal route with 4 x 10^2^ pfu and treated either immediately or 1 or 3 dpi with 150 mg/kg of favipiravir in 0.5% methylcellulose twice daily. Control animals received the same amount of 0.5% methylcellulose without the drug. Weight change and survival were monitored for 14 days after BRBV-STL infection.

### Type I IFN receptor blocking antibody

A type I IFN receptor blocking antibody, MAR1-5A3 (Leinco Technologies, Inc.) or isotype control (2 μg per mouse) were administered to mice via intraperitoneal route prior to inoculation with BRBV-STL.

### RNA *in situ* hybridization assay

RNA in situ hybridization for BRBV was applied to different organs of BRBV-STL infected WT and *Ifnar1*^*-/-*^ mice. Three and six days after subcutaneous infection with 4 x 10^4^ pfu of BRBV-STL, the liver, spleen, heart, brain, lung, and kidney were collected and fixed in 10% formalin for 7 days. Sections of each of these tissues were used for RNA-ISH (ACDBio). Probes against segment 5 of BRBV-STL (NP) were developed by ACDBio and used according to the manufacturers’ recommendations. BRBV-STL positive cells were visualized with 3, 3'-diaminobenzidine (dark brown stain).

### Statistical analysis

Statistical analyses were performed using GraphPad Prism 8.0 software. Differences in mortality were determined using the log-rank (Mantel-Cox) test. Independent t-test with a Holm-Sidak correction for multiple comparisons was used determine statistical significance in weight loss 8 days after BRBV infection. A one-way ANOVA with a Dunnett correction for multiple comparisons was used to determine statistical significance of the effects of favipiravir on polymerase gene activity and cytotoxicity assay.

### Ethics statement

The study was approved by the institutional review board of Washington University School of Medicine in St Louis. Written consent was obtained from the guardian of the patient. Animal experiments were approved and performed in accordance with the recommendations in the Guide for the Care and Use of Laboratory Animals of the National Institutes of Health. The protocols were approved by the Institutional Animal Care and Use Committee at the Washington University School of Medicine (Assurance number A3381-01).

## Supporting information

S1 Fig Histopathology in liver, spleen, kidney, lung, heart and brain of Bourbon virus-infected *Ifnar1*^-/-^ mice. Animals were infected 4 x 10^4^ pfu of BRBV-STL intraperitoneal and with liver, spleen, kidney, lung, heart and brain were collected at 3 (n = 3) and 6 (n = 3) dpi. Organs from an uninfected *Ifnar1*^-/-^ animal (n = 1) were used as a control. Sections were stained with H&E and representative images were collected for each animal. Scale bar is 100 μm.
(TIF)

S2 Fig BRBV RNA detection in liver, spleen, kidney, lung, heart and brain of Bourbon virus-infected *Ifnar1*^-/-^ mice. Animals were infected 4 x 10^4^ pfu of BRBV-STL intraperitoneal and with liver, spleen, kidney, lung, heart and brain were collected at 3 (n = 3) and 6 (n = 3) dpi. Organs from an uninfected *Ifnar1*^-/-^ animal (n = 1) were used as a control. Sections were stained using a RNA probe against segment 5 of BRBV-STL using the ACDbio RNA *in situ* hybridization assay. Viral RNA is indicated by the dark brown stain. Viral RNA is detectable at 3 dpi in the liver and spleen of the animals and at 6 dpi in liver, spleen, kidney, lung and heart of all three animals. Minimal staining was observed in sections of the brain. Representative images were collected for each animal. Scale bar is 100 μm. NA = not available.
(TIF)

S3 Fig Blocking the type I IFN receptor with an antibody (MAR1-5A3) did not result in fatal BRBV disease. Animals received a single dose of the type I IFN receptor blocking antibody MAR1-5A3 (2 μg per mouse, n = 9) or isotype control (2 μg, n = 7) via intraperitoneal route before the animals were inoculated with 4 x 10^4^ pfu of BRBV-STL via the footpad. (A) Mortality was monitored for 20 days and no significant difference was observed between MAR1-5A3 and isotype treated mice. The data are from two different experiment including both isotype and MAR1-5A3 treated animals. (B) Spleen viral load was measured three days after inoculation with 4 x 10^4^ pfu of BRBV-STL via the footpad in three animals per treatment group. A small amount of infectious BRBV was detected in the spleen of the MAR1-5A3 treated animals. Each data point is a single mouse obtained from one experiment.
(TIF)

S4 Fig Favipiravir inhibits BRBV-STL replication in 293T cells. Confluent monolayers of 293T cells in 24-well plates were inoculated with 20 pfu (Multiplicity of infection (MOI) = 0.001) for 1 h at 37°C/5% CO_2_. Next, the inoculum was aspirated and the cells were washed with medium before 1.0 mL of fresh medium with 2% FBS was added to each well. To test the effects of favipiravir, different concentrations (100 μg/mL to 1 μg/mL) of the compound, diluted in DMSO, were added to the wells. Control wells were treated with the same concentration of DMSO. Culture supernatant was collected three days after infection and the amount of infectious virus produced was quantified by plaque assay. The results are the average viral load of one experiment with two wells per condition.
(TIF)
