# Supplementary figures and images for: Therapeutic efficacy of favipiravir against Bourbon virus in mice
Source: PLoS Pathog. 2019 Jun 13;15(6):e1007790. doi: 10.1371/journal.ppat.1007790 (PMC6564012; doi:10.1371/journal.ppat.1007790)

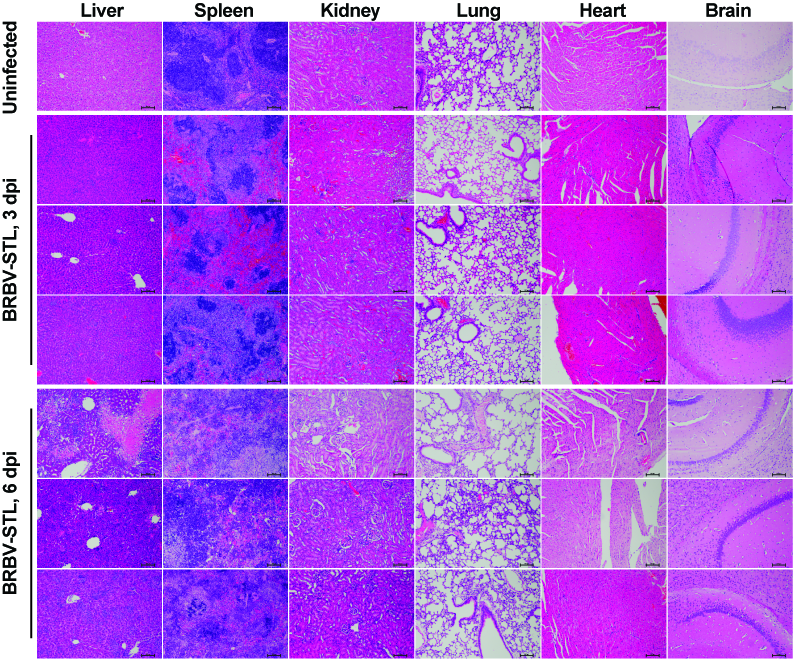

Supplement: S1 Fig — Animals were infected 4 x 10^4^ pfu of BRBV-STL intraperitoneal and with liver, spleen, kidney, lung, heart and brain were collected at 3 (n = 3) and 6 (n = 3) dpi. Organs from an uninfected Ifnar1-/- animal (n = 1) were used as a control. Sections were stained with H&E and representative images were collected for each animal. Scale bar is 100 μm. (TIF) [file ppat.1007790.s001.tif]

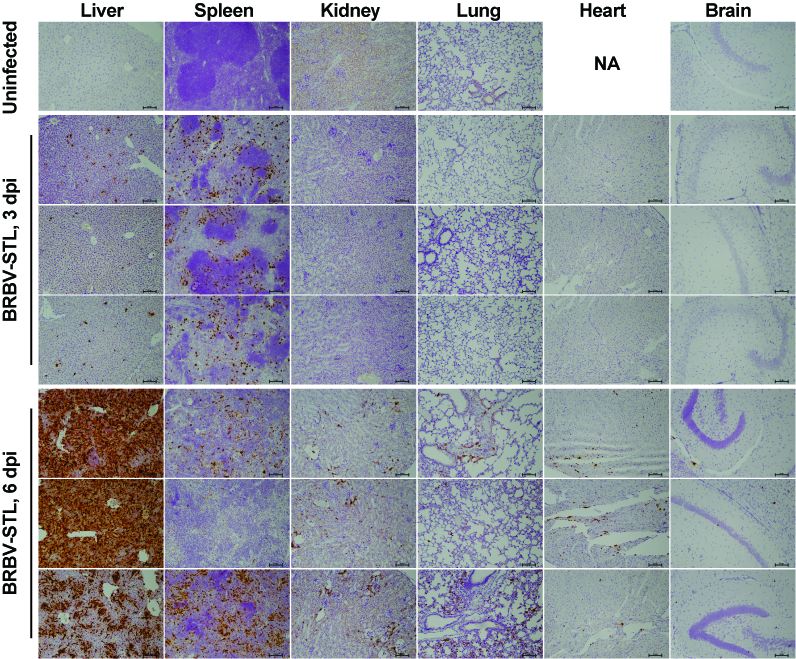

Supplement: S2 Fig — Animals were infected 4 x 10^4^ pfu of BRBV-STL intraperitoneal and with liver, spleen, kidney, lung, heart and brain were collected at 3 (n = 3) and 6 (n = 3) dpi. Organs from an uninfected Ifnar1-/- animal (n = 1) were used as a control. Sections were stained using a RNA probe against segment 5 of BRBV-STL using the ACDbio RNA in situ hybridization assay. Viral RNA is indicated by the dark brown stain. Viral RNA is detectable at 3 dpi in the liver and spleen of the animals and at 6 dpi in liver, spleen, kidney, lung and heart of all three animals. Minimal staining was observed in sections of the brain. Representative images were collected for each animal. Scale bar is 100 μm. NA = not available. (TIF) [file ppat.1007790.s002.tif]

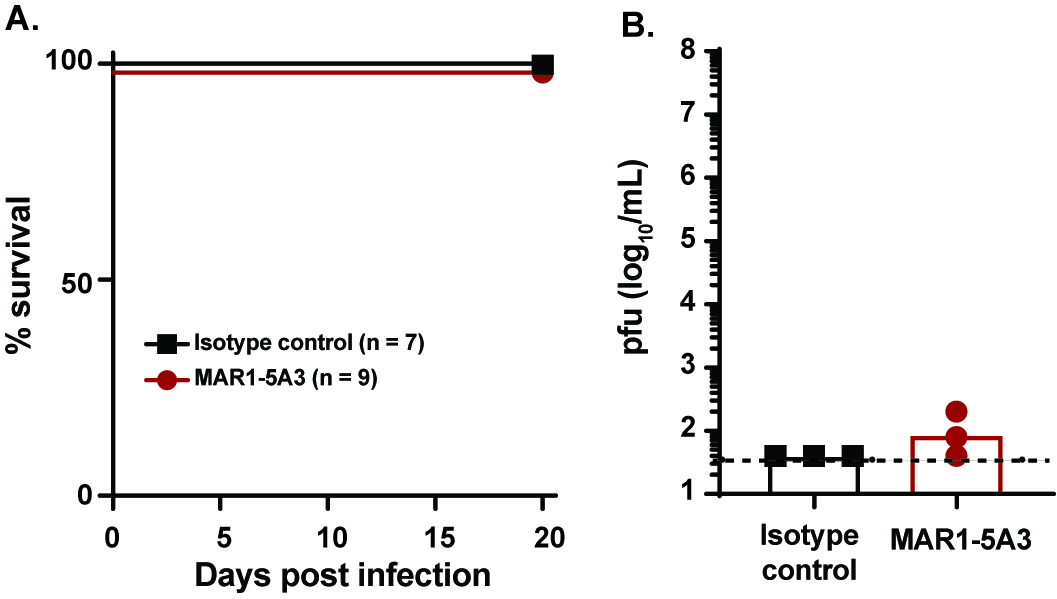

Supplement: S3 Fig — Animals received a single dose of the type I IFN receptor blocking antibody MAR1-5A3 (2 μg per mouse, n = 9) or isotype control (2 μg, n = 7) via intraperitoneal route before the animals were inoculated with 4 x 10^4^ pfu of BRBV-STL via the footpad. (A) Mortality was monitored for 20 days and no significant difference was observed between MAR1-5A3 and isotype treated mice. The data are from two different experiment including both isotype and MAR1-5A3 treated animals. (B) Spleen viral load was measured three days after inoculation with 4 x 10^4^ pfu of BRBV-STL via the footpad in three animals per treatment group. A small amount of infectious BRBV was detected in the spleen of the MAR1-5A3 treated animals. Each data point is a single mouse obtained from one experiment. (TIF) [file ppat.1007790.s003.tif]

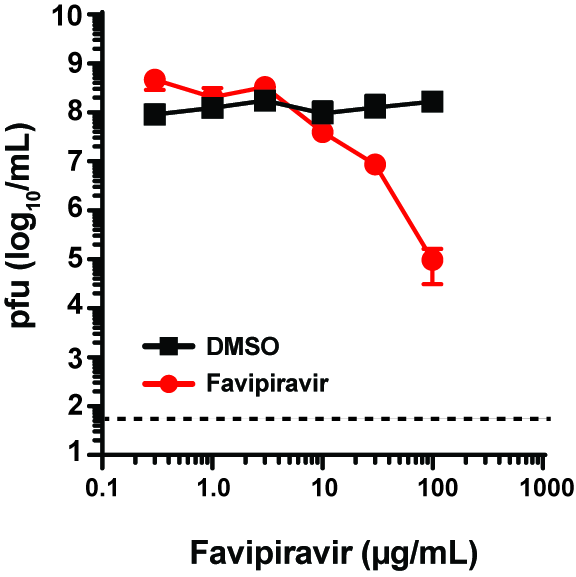

Supplement: S4 Fig — Confluent monolayers of 293T cells in 24-well plates were inoculated with 20 pfu (Multiplicity of infection (MOI) = 0.001) for 1 h at 37°C/5% CO2. Next, the inoculum was aspirated and the cells were washed with medium before 1.0 mL of fresh medium with 2% FBS was added to each well. To test the effects of favipiravir, different concentrations (100 μg/mL to 1 μg/mL) of the compound, diluted in DMSO, were added to the wells. Control wells were treated with the same concentration of DMSO. Culture supernatant was collected three days after infection and the amount of infectious virus produced was quantified by plaque assay. The results are the average viral load of one experiment with two wells per condition. (TIF) [file ppat.1007790.s004.tif]
